# Supplementary material for: Effect of a Multi-Dimensional and Inter-Sectoral Intervention on the Adherence of Psychiatric Patients
Source: PLoS One. 2015 Oct 5;10(10):e0139302. doi: 10.1371/journal.pone.0139302 (PMC4593549; doi:10.1371/journal.pone.0139302)
Supplement: S1 File — (PDF) [file pone.0139302.s001.pdf]

An die Geschäftsstelle der
Ethik-Kommission
der Medizinischen Fakultät
der FAU Erlangen-Nürnberg
Krankenhausstr. 12
91054Erlangen

Antrag
an die Ethik-Kommission
der Medizinischen Fakultät

*Bitte **in deutscher Sprache** ausfüllen,*
Zutreffendes bitte ankreuzen.
Für multizentrische Studien mit Vorvotum einer nach Landesrecht gebildeten zuständigen Ethik-Kommission können Sie das verkürzte Antragsformular verwenden, abzurufen unter: http://www.ethik.med.uni-erlangen.de *(Anschlussvotum)*

Antrag auf Beurteilung eines Forschungsprojektes
(keine Arzneimittelprüfung)

bitte 9-fach einschließlich Anlagen einreichen sowie 1-mal in elektronischer Fassung

Titel des Projektes:

Pharmazeutische Betreuung von psychiatrischen Patienten auf Station

I. Projektleitung

1. Name der/des verantwortlichen Projektleiterin/s an der FAU:
Prof. Dr. Johannes Kornhuber
Direktor der Psychiatrischen und Psychotherapeutischen Klinik
App.-Nr.: 0.853
Telefon: 09131 / 85-34166
E-Mail: johannes.kornhuber@uk-erlangen.de

Angaben über die Qualifikation der/des Versuchsleiterin/s:
Direktor der Psychiatrischen und Psychotherapeutischen Klinik

2. a) Weitere Teilnehmer/innen vor Ort:
Prof. Dr. Kristina Leuner
Professur für Molekulare und Klinische Pharmazie
App.-Nr.: 0.624
Telefon: 09131 / 85-29550
E-Mail: leuner@pharmtech.uni-erlangen.de

Anne Pauly, Apothekerin
Professur für Molekulare und Klinische Pharmazie
App.-Nr.: 0.632
Telefon: 09131 / 85-29575
E-Mail: pauly@pharmtech.uni-erlangen.de

Carolin Wolf, Apothekerin
Professur für Molekulare und Klinische Pharmazie
App.-Nr.: 0.632
Funk: 09131 / 85-29575
E-Mail: cwolf@pharmtech.uni-erlangen.de

b) Weitere Prüfzentren (bei multizentrischen Studien): keine

3. Handelt es sich bei diesem Antrag um ein bereits von der Ethik-Kommission der Medizinischen Fakultät der Friedrich-Alexander-Universität Erlangen-Nürnberg begutachtetes Projekt?
☐ ja (bitte lfd. Nr. angeben) X nein

II. Forschungsvorhaben

1. geplanter Beginn der Studie: 01.September 2012
voraussichtliches Ende: 31.Januar 2014
Dauer der Studienteilnahme für den einzelnen Probanden: 111 Tage (im Durchschnitt 21 Tage stationär, sowie Nachbetreuung für 3 Monate nach Entlassung)

2. Kurzer Abriss des Projektes:
Ziel des geplanten Projekts ist es, in einer klinischen Studie zu erfassen, ob eine intensive Medikamenten-Anamnese sowie eine intensive Beratung des Patienten hinsichtlich seiner Medikation und seiner Erkrankung durch Integration eines Stationsapotheker in das multidisziplinäre Behandlungsteam der Psychiatrischen und Psychotherapeutischen Klinik zu einer Verbesserung bzw. Reduktion folgender Parameter führen kann:

- Anzahl Arzneimittel-bezogener Probleme (AbP) (primärer Endpunkt)
- Medikamentenadhärenz, vor allem nach Entlassung (primärer Endpunkt)

Arzneimittel-bezogene Probleme (AbP) sind definiert als Ereignisse oder Umstände bei der Arzneimitteltherapie des Patienten, die tatsächlich oder potenziell das Erreichen von angestrebten Therapiezielen verhindern [1]. In dieser Studie wird der Fokus auf folgende AbP gelegt: Kongruenz Diagnose und Indikation, Dosierungsfehler, Interaktionen und Nebenwirkungen wie zum Beispiel Gewichtszunahme und sexuelle Funktionsstörung.
Die WHO definiert die Compliance oder Therapietreue als “das Ausmaß, in dem das Verhalten eines Patienten in Bezug auf Arzneimitteleinnahme, Befolgen eines Ernährungsplans oder Anpassungen der Lebensweise mit den Empfehlungen eines Heilberuflers übereinstimmt.” [2]. Unter Adherence oder Adhärenz versteht man eine Erweiterung der Compliance-Definition um die Einbeziehung der Wünsche des Patienten in seine Therapie.

Als sekundäre Endpunkte werden erfasst:

- Einstellung des Patienten gegenüber seinen Medikamenten
- Einfluss der Einstellung des Patienten zu seinen Medikamenten auf die Medikamentenadhärenz

Weitere zu erfassende Parameter:

- Patientenzufriedenheit mit der medizinisch-pharmazeutischen Betreuung

Bei der geplanten Studie zur pharmazeutischen Betreuung handelt es sich um eine prospektive, offene Interventionsstudie mit sequenziellem Kontrollgruppendesign. Eingeschlossen werden psychiatrische Patienten der Stationen P21 und P31 der Psychiatrischen und Psychotherapeutischen Universitätsklinik Erlangen unabhängig ihrer Diagnose.

Weitere Einschlusskriterien müssen erfüllt sein:
- Alter: ≥ 18
- Fähigkeit, selbstständig Fragebögen auszufüllen
- Fähigkeit, gesprochenes und geschriebenes Deutsch zu verstehen
- Einwilligungsfähigkeit
- Stationärer Aufenthalt in der Psychiatrischen Klinik Erlangen > 7 Tage
- Bereitschaft zur Kontaktaufnahme nach Entlassung

Ausgeschlossen werden Patienten, die ausschließlich psychotherapeutisch behandelt werden und keine zusätzliche Begleitmedikation (nicht-psychiatrisch) einnehmen. Patienten, die von der geschützten Frauen-Station P12 auf die teilnehmenden, offenen Stationen verlegt werden, werden außerdem ausgeschlossen.

Bei Aufnahme ins Krankenhaus erfolgt eine ausführliche mündliche und schriftliche Aufklärung der Patienten über das geplante Projekt. Anschließend erhalten die Patienten schriftliches Informationsmaterial sowie das Formblatt zur Einverständniserklärung. Nach Ablauf einer Bedenkzeit von mindestens 24 Stunden wird das Formblatt wieder eingesammelt und der Patient entsprechend seiner Entscheidung in die Studie eingeschlossen oder nicht.
Um eine Überlappung von betreuten und nicht betreuten Patienten auf den Stationen zu verhindern, wird eine sequenzielle Kontrollgruppe verwendet: In den ersten 6 Monaten (KW 36/2012 – 09/2013) der Studie werden Patienten nur in die Kontrollgruppe aufgenommen, in den nächsten 6 Monaten der Studie (KW 14/2013 - KW 39/2013) werden die Patienten dann nur in die Interventionsgruppe aufgenommen. Zwischen den Gruppenphasen erfolgt eine Wash-Out-Periode von 4 Wochen, um sicher zu stellen, dass die Patienten der Kontrollgruppe die stationäre Studienphase vor Beginn der Interventionsphase komplett beendet haben.

Ablauf der Betreuung:
1-2 Tage nach Aufnahme in die Klinik erfolgt bei allen Studienteilnehmern eine gründliche Medikamentenanamnese durch die Apothekerinnen. Jede Verordnung wir mit Hilfe des „Medication Appropriateness Index“ geprüft [3]. Bei allen Patienten werden Nebenwirkungen und die Adhärenz mit Hilfe von validierten Fragebögen erfasst

(siehe dazu 3. Studienbezogene Maßnahmen). Diese Fragebögen füllen die Patienten zusammen mit den Apothekerinnen 1-4 Tage nach Aufnahme, einen Tag vor und drei Monate nach Entlassung aus. Diese Ergebnisse werden zusammen mit dem Medikationsprofil auf AbP wie zum Beispiel fehlende Indikation und Dosierungsprobleme untersucht. Besonderes Augenmerk liegt hierbei auf der Ermittlung von Wechselwirkungen mit der Interaktionsdatenbank „Stockleys."
In der Kontrollgruppe werden die AbP nur identifiziert und dokumentiert (Ausnahme aus ethischen Gründen: lebensbedrohliche oder massiv krankheitsverschlechternde Komplikationen).
In der Interventionsgruppe soll dagegen in Zusammenarbeit mit dem Arzt eine Lösung der AbP erarbeitet werden.
Die Adhärenz wird in beiden Gruppen während des Klinikaufenthaltes (1-4 Tage nach Aufnahme, sowie 1-2 Tage vor der Entlassung) und 3 Monate nach Entlassung erfasst.
Patienten der Interventionsgruppe erhalten nach ca. 10 Tagen Klinikaufenthalt eine intensive Beratung über ihre Erkrankungen sowie eine ausführliche Aufklärung über die einzunehmenden Arzneimittel, deren Nutzen und Nebenwirkungen.
Da die Adhärenz vor allem nach der Entlassung gefährdet ist, erhalten Patienten der Interventionsgruppe einen Medikationsplan sowie einen ausführlichen Brief zur Entlassmedikation für den betreuenden niedergelassenen Psychiater/Hausarzt. Darüber hinaus wird diesen Patienten während der folgenden 3 Monate nach Entlassung aus der Klinik ein telefonisches Follow-up-Gespräch zur Adhärenzverbesserung durch eine Apothekerin angeboten. In diesem Telefongespräch soll nach Umstellung der Medikation durch den weiterbetreuenden Arzt, der Zufriedenheit mit der Therapie und der Adhärenz gefragt werden. Offene Fragen und individuelle Probleme der Patienten mit den Arzneimitteln können geklärt werden. Wenn nötig, wird eine ausführliche Beratung durchgeführt. Somit soll durch die medizinisch-pharmazeutische Betreuung die Schnittstelle zwischen stationärer und ambulanter Behandlung geschlossen werden.

Eine Übersicht über den zeitlichen Ablauf der Studie befindet sich in Anhang Nr. 1.

3. **Studienbezogene Maßnahmen:**

Bitte beschreiben Sie hier alle Maßnahmen, die studienbedingt durchgeführt werden sowie alle erforderlichen Abweichungen von der üblichen Routine-Behandlung:

Zur Erfassung der oben genannten Parameter füllen die Patienten der Interventions- und Kontrollgruppe folgende validierte Fragebögen in Zusammenarbeit mit der Apothekerin bei Aufnahme, ca. 1-2 Tage vor Entlassung und in der Nachbetreuungsphase ca. 3 Monaten nach Entlassung aus:

Fragebogen Patient		Zu erfassende Parameter
**UKU-SERS**	The Udvalg for Kliniske Undersøgelser Side Effect Rating Scale, nach Lingjaerde et al	Nebenwirkungen psychotroper Medikamente

**MARS-D / DAI**	Medication Adherence Rating Scale- deutsche Version, nach Mahler et al<br>Drug Attitude Inventory, nach Hogan et al	Medikamentenadhärenz / Einstellung gegenüber Medikamenten
**BphB (nur Interventionsgruppe)**	Beurteilung des pharmazeutischen Betreuungsservice	Zufriedenheit mit der pharmazeutischen Betreuung

Als Leitfaden zur Identifikation Arzneimittel-bezogener Probleme, sowie zum Vergleich von Aufnahme- und Entlassmedikation in Hinblick auf Angemessenheit der Therapie wird der „Medication Appropriateness Index“ von den Apothekerinnen ausgefüllt.

Weiterhin wird eine Beratung über die einzunehmenden Medikamente und die diagnostizierten Erkrankungen für die Patienten der Interventionsgruppe angeboten. Die Beratung beinhaltet ausführliche Informationen über:

- Psychiatrische Erkrankung des Patienten
- Begleiterkrankungen (z.B. Hypertonie, Diabetes mellitus)
- Welches Medikament für welche Erkrankung indiziert ist.
- Nebenwirkungen und besondere Wirkprofile, Wechselwirkungen
- Dosierungs- und Einnahmeregimes

Die Adhärenzsicherung bei Entlassung des Patienten von der Klinik in den ambulanten Bereich steht dabei im Vordergrund (Schnittstellenmanagement).
Die Patientenzufriedenheit mit der pharmazeutischen Betreuung wird mit einem Fragebogen beurteilt, der für eine pharmazeutische Betreuungsstudie an Bypass-Patienten entwickelt worden ist. [9]

4. Wird die Studie gemäß der von der 48. Generalversammlung des Weltärztebundes in Somerset West revidierten Deklaration von Helsinki aus dem Jahre 1996 durchgeführt?
JA
Bitte angeben, ob alle anderen Erprobungsmöglichkeiten ausgeschöpft wurden.
Keine weiteren Erprobungsmöglichkeiten bekannt

5. Art des Forschungsvorhabens:
Handelt es sich um
☐ eine diagnostische Prüfung?
☐ eine therapeutische Prüfung?
☐ eine Verträglichkeitsprüfung?
☐ einen ausschließlich wissenschaftlichen Versuch?
X eine Studie zur Versorgungsforschung

6. Gesetzliche Grundlagen
a) Handelt es sich um eine Untersuchung, die dazu bestimmt ist, klinische oder pharmakologische Wirkungen von Arzneimitteln zu erforschen oder nachzuweisen oder Nebenwirkungen festzustellen oder die Resorption, die Verteilung, den Stoffwechsel oder die Ausscheidung zu untersuchen, **mit dem Ziel, sich von der**

Unbedenklichkeit oder Wirksamkeit des Arzneimittels zu überzeugen (klinische Prüfung eines Arzneimittels nach §§ 40 Arzneimittelgesetz)?

Nein, pharmazeutische Betreuungsstudie.

b) Handelt es sich um eine klinische Prüfung nach § 20 Medizinproduktegesetz (MPG)?
 ja X nein

Bitte begründen. Liegt eine CE-Zertifizierung für das Medizinprodukt vor? Werden zusätzlich invasive oder andere belastende Untersuchungen durchgeführt?

c) Handelt es sich um ein Vorhaben nach § 8 des Gesetzes zur Regelung des Transfusionswesens (TFG)?
 ja X nein

7. Handelt es sich um einen Versuch nach
§ 23 Strahlenschutzverordnung? ja X nein
§ 28 Röntgenverordnung? ja X nein

Bitte begründen. Falls ja: *Werden die hiernach erforderlichen behördlichen Gutachten eingeholt?*

8. Typ der Studie:
X offen
 blind
 doppelblind
X vergleichend
 randomisiert
 multizentrisch
 Feldstudie
X Pilotstudie

9. Wissenschaftliche Begründung des Projekts, insbesondere:

a. Erläuterung des Versuchsziels
Primärziele der Studie sind zum einen die Reduktion von AbP und zum anderen die Verbesserung der Adhärenz durch medizinisch-pharmazeutische Betreuung im stationären Setting sowie vor allem 3 Monate nach Entlassung.

b. Darstellung des bisherigen Wissensstandes
Die 12-Monats-Prävalenz psychiatrischer Erkrankungen liegt in Deutschland für Frauen bei 37% und für Männer bei 25,3% [4]. Sie hat in den letzten Jahren, vor allem für Depressive Erkrankungen, zugenommen [5]. Eine genaue und vollständige Arzneimittelanamnese ist bei dieser Patientengruppe enorm wichtig, da psychiatrische Erkrankungen häufig von Komorbiditäten und damit von Polypharmazie begleitet werden [6]. Das Risiko für AbP steigt damit exponentiell an. Weiterhin ist durch die spezielle Wirkweise vieler psychotroper Medikamente (Wirkungseintritt erst nach 2-3 Wochen, Auftreten von Nebenwirkungen jedoch sofort) die Rate der Therapieabbrüche sehr hoch. Die Anzahl adhärenter Patienten schwankt auch nach Einführung der modernen Präparate zwischen 20-70% [2]. Die Adhärenz stellt jedoch ein sehr wichtiges Standbein einer adäquaten und

erfolgreichen Therapie psychischer Erkrankungen dar. Das Risiko einer erneuten Krankenhauseinweisung ist nach Valenstein et al für einen nicht-adhärenten schizophrenen Patienten im Vergleich zu einem adhärenten schizophrenen Patienten um das 2,4fache erhöht [7]. Ähnliches gilt für Patienten mit affektiven Störungen. Ein vorzeitiger Therapieabbruch führt bei affektiven Störungen zu einer 77%igen Erhöhung des Rückfallrisikos [8].

Ein Ansatz zur Reduktion von AbP sowie zur Verbesserung der Adhärenz ist das Konzept der medizinisch-pharmazeutischen Betreuung. Medizinisch-pharmazeutische Betreuung basiert auf der intensiven Zusammenarbeit zwischen Patient, behandelndem Arzt und Apotheker. Ziel ist es, den Patienten gemeinsam mit dem behandelnden Arzt zu einer sicheren und sachgerechten Arzneimittelanwendung anzuleiten, mögliche AbP zu erkennen und zu lösen, die Arzneimitteltherapie zu optimieren und daraus resultierend die Lebensqualität des Patienten zu verbessern.

Eine niederländische Arbeitsgruppe um Joanna Klopotowska untersuchte den Einfluss eines Stationsapothekers auf die Anzahl AbP auf einer Intensivstation. Dabei konnte eine Reduktion der AbP von 190,5 pro 1000 beobachteten Patiententagen auf 62,5 pro 1000 beobachteten Patiententagen während der Interventionsphase nachgewiesen werden [9]. In einer Studie zur Pharmazeutischen Betreuung von Patienten mit koronarer Herzkrankheit nach aortokornarer Bypassoperation am Universitätsklinikum Erlangen konnte eine signifikante Adhärenzsteigerung gezeigt werden. Nach 12 Monaten lag die Adhärenz in der Interventionsgruppe bei 90% (Basiswert: 77%) und in der Kontrollgruppe bei 71% (Basiswert 81%). Das Betreuungskonzept umfasste neben Gesprächen mit einem Krankenhausapotheker während des stationären Aufenthaltes auch Follow-up-Gespräche bei Ambulanzbesuchen und beinhaltete die Aushändigung schriftlicher Unterlagen [10].

Mit der vorliegenden geplanten Studie zur pharmazeutischen Betreuung von psychiatrischen Patienten auf Station, soll untersucht werden, ob sich ähnlich positive Effekte auf die Anzahl der AbP, sowie die Medikamentenadhärenz dieser Patientengruppe erzielen lassen. Zur Quantifizierung dienen hierbei unter anderem validierte Fragebögen.

Unseres Wissens nach gibt es in Deutschland bisher keine vergleichbaren Studien mit psychiatrischen Patienten. Ein erstes Projekt in Großbritannien erfasste 282 Interventionen durch den Apotheker auf Station [11]. Eine Betreuungsstudie in den USA brachte bei 62% der Patienten eine mehr als 30%ige Verbesserung im Brief Psychiatric Rating Scale [12]. In einer weiteren amerikanischen Studie wurde eine Verbesserung der Adhärenz um 19% erreicht [13]. Mit der vorliegenden, geplanten Studie soll erstmals medizinisch-pharmazeutische Betreuung durch Stationsapotheker in enger Kooperation mit den Klinikärzten auf einer deutschen, psychiatrischen Station implementiert, aber auch gleichzeitig der Nutzen der geleisteten Betreuung des Apothekers quantitativ auswertbar dargestellt werden.

10. Angaben zur Nutzen-Risiko-Relation

a. Welcher Nutzen ist von den Ergebnissen der Studie zu erwarten

aa) für die Versuchsteilnehmer?

Durch eine gründliche Anamnese sollen AbP wie z.B. Doppelverordnungen, Interaktionen und überflüssige Arzneimitteleinnahmen verhindert werden, um

damit das Behandlungsregime für den Patienten so effektiv und einfach wie möglich zu gestalten.
Zudem wird der Therapieerfolg, also die Remission der Erkrankung, auch über einen längeren Zeitraum, ganz entscheidend von der Adhärenz des Patienten beeinflusst. Ziel dieses Projekts ist es, durch konsequente Schulung und Beratung zur ordnungsgemäßen Arzneimittelanwendung das Verständnis über Medikation und Erkrankung zu verbessern, um die Adhärenz zu erhöhen. Mehrfachgaben von Präparaten sollen, wenn möglich, durch Einmaldosen (Retardpräparate) ersetzt werden, um das Therapieschema der Patienten zu vereinfachen. Durch Aufklärung zu Vermeidungsstrategien für potentielle Nebenwirkungen und Behandlung von bzw. Umgang mit tatsächlich auftretenden Nebenwirkungen soll die Patientenzufriedenheit mit der Behandlung erhöht und damit ebenfalls ein wichtiger Beitrag zur Therapietreue geleistet werden.

ab) für die Heilkunde?
Doppelverordnung, Verschreibungen ohne Indikation oder auch unerwünschte Arzneimittelwirkungen durch Interaktionen können durch eine gezielte Anamnese verhindert und damit verbundene arzneimittelbezogene Kosten eingespart werden. Weiterhin führt ein langfristiger Therapieerfolg zu einer reduzierten Rezidivrate und damit zu einer reduzierten Rehospitalisierungsrate. Somit stellt die medizinisch-pharmazeutische Betreuung bei positiven Studienergebnissen einen Ansatz zur Verbesserung der Versorgungsqualität sowie zum wirtschaftlichen Ressourceneinsatz dar.

ac) für die Wissenschaft (z.B. Ergebnisse, die nicht unmittelbar therapeutischen Zwecken dienen)?
Erstmalig versucht ein Apotheker in Deutschland auf einer psychiatrischen Station durch medizinisch-pharmazeutische Betreuung AbP zu reduzieren und die Patientenadhärenz zu verbessern, um somit den Therapieerfolg zu unterstützen. Durch das Design der Studie mit Kontrollgruppe kann erstmals die Reduktion der Arzneimittel-bezogenen Probleme im Vergleich zur Standardbetreuung aufgezeigt werden. Zusätzlich soll der Einfluss einer pharmazeutischen Nachbetreuung der Patienten auf die Adhärenz nach Entlassung aus der Klinik mit Daten belegt werden.

b. **Mit welchem Risiko ist die Studie für die Versuchsteilnehmer verbunden?**

ba) Welcher Art sind die Risiken? Risikoeinschätzung, vorhersehbare Risiken der Behandlung und sonstiger studienbedingter Verfahren, die eingesetzt werden sollen (einschließlich Schmerz, Unannehmlichkeiten, Beschwerden, Verletzung der persönlichen Integrität und Maßnahmen zur Vermeidung und/oder zur Behandlung von unvorhersehbaren/ unerwünschten Ereignissen)
Die Studie ist mit keinem Risiko verbunden.

bb) Mit welcher Wahrscheinlichkeit ist zu erwarten, daß sich die Risiken realisieren? Wie sicher ist die Wahrscheinlichkeit abschätzbar? /

c. **Warum ist das mögliche Risiko im Verhältnis zu dem zu erwartenden Nutzen Ihrer Ansicht nach vertretbar?**
/

d. Werden Zwischenergebnisse ausgewertet, um einen Trend zu erkennen?

X ja ☐ nein

e. Sind Kriterien festgelegt worden, bei deren Eintreten der Versuch geändert oder abgebrochen werden soll? ☐ ja, welche? X nein

11. Bei klinischen Prüfungen nach §§ 20-24 MPG:
/

Bitte fügen Sie die gemäß § 3 Abs. 1-3 der Verordnung über klinische Prüfungen von Medizinprodukten und zur Änderung medizinprodukterechtlicher Vorschriften (MPKPV) *erforderlichen Anlagen bei. Die aktuelle Fassung der MPKPV können Sie über die Homepage der Ethik-Kommission http://www.ethik.med.uni-erlangen.de abrufen.*

12. a) Ist die Mitarbeit eines Statistikers vorgesehen? X ja ☐ nein
b) Welche statistischen Methoden sollen benutzt werden?
Mit Ausnahme der Patientenzufriedenheit mit der pharmazeutischen Betreuung (nur deskriptiv) werden für alle Messwerte (primäre und sekundäre Endpunkte) Mittelwert bzw. Median, Wertebereiche, Standardabweichung und Konfidenzintervall angegeben. Zum Nachweis statistisch bedeutsamer Unterschiede werden für kontinuierliche Messwerte bei vermuteten normalverteilten Werten der t-Test, bei vermuteten nicht-normalverteilte Werten der Mann-Whitney-U-Test durchgeführt. Bei binären Werten wird der Chi-Quadrat-Test durchgeführt.

13. a) Handelt es sich um eine multizentrische Studie (d.h. eine nach einem *einzigen* Prüfplan durchgeführte Studie, die in mehr als einer Prüfstelle erfolgt und daher von mehr als einem Prüfer vorgenommen wird)? ☐ ja X nein

b) Wurden/Werden an anderer Stelle Studien mit demselben oder einem ähnlichen Ziel durchgeführt? ☐ ja, wo? X nein

14. Wer hat die Studie initiiert?
Psychiatrische und Psychotherapeutische Klinik und Professur für Molekulare und Klinische Pharmazie

15. Wer finanziert sie? (*Bitte geben Sie an, ob Drittmittel von nichtöffentlicher Seite beantragt werden. Falls ja, in welcher Höhe?*)
Die Studie wird im Rahmen zweier Promotionsprojekte entwickelt und durchgeführt.

16. Die Aufwandsentschädigung für die Begutachtung wird übernommen von (*bitte Ansprechpartner benennen*):
Professur für Molekulare und Klinische Pharmazie (Prof. Dr. Kristina Leuner)

III. Angaben zu den Versuchsteilnehmern

1. Anzahl (*bei vergleichenden Studien bitte Aufteilung auf Gruppen angeben*)
Einschluss von je 123 Patienten in der Kontroll- und in der Interventionsgruppe

Bei Nullhypothesen-basierten Studien:
Wurde eine formale Fallzahlschätzung vorgenommen?
X ja ☐ nein

2. Alter und Geschlecht (*bitte geben Sie das Alter der Versuchsteilnehmer sowie die als Ausschlusskriterien vorgesehenen Ober- und Untergrenzen an*)
Alter: 18 - ∞ Jahre, beide Geschlechter

3. Status: Handelt es sich bei den Versuchsteilnehmern um
☐ gesunde Personen
☐ schwangere oder stillende Frauen
☐ Kinder oder Jugendliche
X einschlägig Erkrankte mit psychischen Erkrankungen jeglicher ICD-10 Diagnosen
☐ Personen, die an anderen Krankheiten leiden? (Insbesondere: psychische Krankheiten, die Zweifel an der Geschäfts- oder Einsichtsfähigkeit begründen)

4. Welche sonstigen **Einschlusskriterien** (z.B. erlaubte Begleitmedikation) sind vorgesehen?
- Alter: ≥ 18
- Fähigkeit, selbstständig Fragebögen auszufüllen
- Fähigkeit, gesprochenes und geschriebenes Deutsch zu verstehen
- Einwilligungsfähigkeit
- Stationärer Aufenthalt in der Psychiatrischen Klinik Erlangen auf den Stationen P21 oder P31 > 7 Tage
- Bereitschaft zur Kontaktaufnahme nach Entlassung

5. Welche sonstigen **Ausschlusskriterien** (z.B. fortgeschrittene Nieren- oder Leber-insuffizienz, verbotene Begleitmedikation etc.) sind vorgesehen?
- Ausschließlich psychotherapeutische Behandlung ohne weitere Begleitmedikation
- Verlegung von geschlossener Frauen-Station P12 auf offene Station

6. Sollen auch Personen teilnehmen, die auf gerichtliche oder behördliche Anordnung in einer Anstalt verwahrt werden?
☐ ja X nein

7. Sollen auch Personen teilnehmen, die sich schon für andere Forschungsvorhaben zur Verfügung gestellt haben?
X ja ☐ nein
wie lange muss die letzte Teilnahme zurückliegen? /

8. Bei Studien an Minderjährigen (oder sonst nicht geschäftsfähigen Personen)
a. Warum kann die Studie nicht an Erwachsenen (voll Geschäftsfähigen) durchgeführt werden?
b. Sind Aufklärung und Einwilligung der (des) gesetzlichen Vertreter(s) gewährleistet?

(*bitte vorformulierte Erklärung beifügen*)
 ja nein, weil

c. Sind zusätzliche Aufklärung und Einwilligung der minderjährigen (nicht voll geschäftsfähigen) Versuchsteilnehmer gewährleistet, die selbst in der Lage sind, Wesen, Bedeutung und Tragweite des Versuchs einzusehen und ihren Willen danach zu bestimmen?
 ja nein

9. Probandenversicherung
Wird zugunsten der Versuchsteilnehmer eine Versicherung abgeschlossen?
 ja (*bitte Police beifügen, aus der die Versicherungsgesellschaft und die Höhe der Versicherungsleistung hervorgeht)*
X nein

10. Schweigepflicht/Datenschutz
Werden die ärztlichen Schweigepflicht- und die Datenschutzbestimmungen beachtet?
JA

11. Entgelt für Probanden
Soll den Versuchsteilnehmern ein Entgelt (Aufwandsentschädigung o.ä.) gezahlt werden?
 ja, in Höhe von EUR X nein

12. Wie sollen die Versuchsteilnehmer über Wesen, Bedeutung und Tragweite der Studie **aufgeklärt** werden?
Bitte in deutscher Sprache beifügen:
Dokumentation des Inhalts der Patientenaufklärung durch die/den versuchsdurchführende/n Ärztin/Arzt (Merkblatt), insbesondere mit Hinweisen über:
- **Ziele und Methoden** der Studie;
- **Nutzen und Risiko** der Studie;
- bekannte und möglicherweise zu erwartende **Wirkungen und Nebenwirkungen** von Medikamenten;
- Eingriffe, die nur aus wissenschaftlichen Gründen erfolgen;
- ein angebrachtes Verhalten des Patienten während und nach dem Versuch;
- die **Widerruflichkeit** einer Einwilligung;
- **Ausschlusskriterien** (z.B. Schwangerschaft/Stillzeit);
- Name und Telefon des **Ansprechpartner**s vor Ort.

Liegt bei (Anhang Nr. 2)!

13. Wie sollen die Versuchsteilnehmer ihre **Einwilligung** in die Teilnahme an der Studie erklären? (*bitte formulierte deutschsprachige Erklärung mit datenschutzrechtlicher Einwilligungserklärung beifügen*)

Liegt bei (Anhang Nr. 3)!

Literatur:

1. Foppe van Mil, J.W., et al., *Drug-related problems in public pharmacies.* Arzneimittelbezogene probleme in der öffentlichen apotheke, 2001. **146**(16): p. 24-30.
2. World-Health-Organization, *Adherence to long-term therapies: evidence for action.* . Geneva, 2003.
3. Hanlon, J.T., et al., *A method for assessing drug therapy appropriateness.* Journal of Clinical Epidemiology, 1992. **45**(10): p. 1045-1051.
4. Jacobi, F., M. Klose, and H.U. Wittchen, *[Mental disorders in the community: healthcare utilization and disability days].* Bundesgesundheitsblatt Gesundheitsforschung Gesundheitsschutz, 2004. **47**(8): p. 736-44.
5. Bühren, A., et al., *Mental disorders: All specialties are needed.* Psychische erkrankungen - Alle fachgebiete sind gefordert, 2008. **105**(17): p. A880-A884.
6. Kampfhammer, H., *Psychische Störungen bei somatischen Krankheiten.* Psychiatrie, Psychosomatik und Psychotherapie, Springer-Verlag, 2011. **4. Auflage**.
7. Valenstein, M., et al., *Pharmacy data identify poorly adherent patients with schizophrenia at increased risk for admission.* Med Care, 2002. **40**(8): p. 630-9.
8. Melfi, C.A., et al., *The effects of adherence to antidepressant treatment guidelines on relapse and recurrence of depression.* Archives of General Psychiatry, 1998. **55**(12): p. 1128-1132.
9. Klopotowska, J.E., et al., *On-ward participation of a hospital pharmacist in a Dutch intensive care unit reduces prescribing errors and related patient harm: an intervention study.* Crit Care, 2010. **14**(5): p. R174.
10. Koch, S., *Pharmazeutische Betreuung von Patienten mit koronarer Herzkrankheit nach aortokoronarer Bypassoperation - eine Pilotsudie -.* Dissertation, 2009.
11. Dolder, C., et al., *Pharmacist interventions in an inpatient geriatric psychiatry unit.* American Journal of Health-System Pharmacy, 2008. **65**(19): p. 1795-1796.
12. Canales, P.L., P.G. Dorson, and M.L. Crismon, *Outcomes assessment of clinical pharmacy services in a psychiatric inpatient setting.* American Journal of Health-System Pharmacy, 2001. **58**(14): p. 1309-1316.
13. Finley, P.R., et al., *Impact of a collaborative care model on depression in a primary care setting: A randomized controlled trial.* Pharmacotherapy, 2003. **23**(9 I): p. 1175-1185.

Ich weiß, daß auch bei einer positiven Beurteilung des Vorhabens durch die Ethik-Kommission der Medizinischen Fakultät der FAU Erlangen-Nürnberg die ärztliche und juristische Verantwortung für die Durchführung des Projektes uneingeschränkt bei der Leiterin/dem Leiter verbleibt.

Erlangen/Nürnberg
Datum 06.07.12

Unterschrift des/der Antragstellers/in

Kornhuber
(Name in Druckbuchstaben)

Unterschrift der/des Leiterin/Leiters der Einrichtung, in der das Vorhaben durchgeführt werden soll.
Mit der Durchführung des Forschungsvorhabens einverstanden:

Datum 06.07.12

Unterschrift des/der Leiters/Leiterin der Einrichtung

Kornhuber
(Name in Druckbuchstaben)

Pharmazeutische Betreuung psychiatrischer Patienten

Patienteninformation

Liebe Patientin, lieber Patient,

Sie wurden vor kurzem in der Psychiatrischen und Psychotherapeutischen Klinik des Universitätsklinikums Erlangen aufgenommen. Wir hoffen, dass Sie sich hier wohlfühlen und bald Besserung finden.

Die Psychiatrische Klinik der Universität Erlangen und die Professur für Molekulare und Klinische Pharmazie führen seit März 2012 ein Projekt zur pharmazeutischen Betreuung psychiatrischer Patienten unter Leitung von Herrn Prof. Kornhuber, Direktor der Psychiatrischen und Psychotherapeutischen Klinik, durch. Dabei wollen wir klären, ob es sinnvoll ist, psychiatrische Patienten neben ihrer bisherigen Therapie zusätzlich durch einen Apotheker zu ihrer Arzneimittelanwendung zu informieren und strukturiert zu betreuen.

Hintergründe und Ziele des Projektes:
Der Erfolg einer psychiatrischen Therapie hängt in hohem Maße von Ihrer Mitarbeit ab. So kann ein Medikament seine Wirkung nur entfalten, wenn es von Ihnen als Patient regelmäßig und richtig eingenommen wird. Ein Ziel unseres Projektes ist es daher, durch unsere pharmazeutische Betreuung Ihre sichere und gute Arzneimittelanwendung zu fördern und gemeinsam mit Ihnen und den behandelnden Ärzten zu einem verbesserten Therapieergebnis beizutragen.
Eine gründliche Überprüfung Ihrer Medikation durch eine Apothekerin kann eventuell auftretende Probleme wie z.B. Wechsel- oder Nebenwirkungen frühzeitig aufdecken und damit verhindern. Als zweites Ziel möchten wir somit erreichen, dass Ihre Arzneimitteltherapie noch sicherer und angenehmer wird.
Langfristig soll durch die intensive Zusammenarbeit zwischen Arzt, Pflege und Apotheker die Qualität der Arzneimitteltherapie im Krankenhaus verbessert werden.

Die beschriebene Studie dient *nicht* dazu, neue Arzneimittel zu testen.

Ablauf des Projektes:
Die Betreuungsdauer für alle Projektteilnehmer erstreckt sich über den gesamten Klinikaufenthalt. Die ambulante Nachbetreuung endet 3 Monate nach Entlassung. Um die Einflüsse der zusätzlichen Betreuung durch einen Apotheker untersuchen zu können, werden die Teilnehmer der Studie nach Aufnahmedatum in folgende zwei Gruppen eingeteilt:

Gruppe 1 (September 2012 – Februar 2013):
Patienten dieser Gruppe erhalten die übliche Betreuung durch das ärztliche und pflegerische Team der Stationen. Während Ihres stationären Aufenthaltes sowie im Rahmen des Gesprächs 3 Monate nach Entlassung aus dem Krankenhaus erhalten Sie drei Fragebögen zu den Themen „Symptome und Nebenwirkungen“, „Arzneimitteleinnahmeverhalten“ und gegebenenfalls „auf das Körpergewicht bezogene Lebensqualität“. Diese werden gemeinsam mit der betreuenden Apothekerin ausgefüllt. Außerdem wird Ihr Medikationsprofil von einer Apothekerin aufgenommen.

Gruppe 2 (April 2013 – September 2013):
Patienten dieser Gruppe erhalten neben der üblichen Betreuung durch das ärztliche und pflegerische Team der Station individuelle Beratungsgespräche sowie eine gründliche Überprüfung des Medikationsprofils durch eine Apothekerin. Sie erhalten eine Woche und drei Monate nach Entlassung einen Termin für ein weiteres Beratungsgespräch mit der betreuenden Apothekerin.
Während Ihres stationären Aufenthaltes sowie im Rahmen der Gespräche nach Entlassung erhalten Sie drei Fragebögen zu den Themen „Symptome und Nebenwirkungen", „Arzneimitteleinnahmeverhalten", „Lebensqualität" sowie einmalig den Fragebogen „Beurteilung des pharmazeutischen Betreuungsservice". Diese Fragebögen werden gemeinsam mit der betreuenden Apothekerin ausgefüllt. Auf Grundlage des Medikationsprofils und der Fragebögen möchten wir die auftretenden Probleme der Patienten mit ihrer Therapie in Zusammenarbeit mit Arzt und Pflege lösen.

Risiken und Nutzen des Projektes:
Die ärztliche Therapie im Rahmen Ihres Aufenthaltes in der Psychiatrischen Klinik wird durch dieses Projekt in keiner Weise beeinflusst. Es entstehen keine zusätzlichen Risiken für Sie. Die endgültige Entscheidung der Arzneimitteltherapie liegt unverändert beim behandelnden Arzt.
Ihre Arzneimitteleinnahme soll für Sie so einfach, angenehm und sicher wie möglich gestaltet werden. Dadurch sollen Therapietreue und Therapieerfolg verbessert sowie eventuell auftretende Probleme reduziert werden.

Datenschutz:
Im Laufe der Studie werden patientenbezogene Daten von Ihnen erfasst. Dabei handelt es sich neben allgemeinen Angaben zu Ihrer Person zum einen um die Dokumentation Ihrer Medikation und zum anderen um Daten aus den von Ihnen ausgefüllten Fragebögen und den mit Ihnen geführten Beratungsgesprächen. Alle anfallenden Daten werden nur verschlüsselt gespeichert und ausgewertet sowie anonymisiert für eine mögliche Publikation verwendet. Die personalisierten Daten werden Dritten nicht zugänglich gemacht.

Rechte der Teilnehmer:
Die Teilnahme an der oben beschriebenen Studie ist freiwillig. Sollten Sie die Teilnahme an der Studie ablehnen, ist Ihr weiterer Therapieablauf in der Psychiatrischen und Psychotherapeutischen Klinik natürlich in keiner Weise beeinträchtigt.
Wenn Sie im Laufe der Studie Ihre Einwilligung zurückziehen möchten, ist dies jederzeit und ohne Angabe von Gründen möglich. Es entstehen Ihnen hierdurch selbstverständlich keinerlei Nachteile.

Wir hoffen, dass wir Ihr Interesse an unserer Studie zur pharmazeutischen Betreuung psychiatrischer Patienten geweckt haben. Wir würden uns freuen, Sie als Teilnehmer/in begrüßen zu dürfen. Zu diesem Zweck ist es notwendig, beiliegende Einverständniserklärung zu unterzeichnen.

Wenn Sie noch weitere Fragen zu unserem Projekt oder dessen Ablauf haben, stehen Ihnen Ihre betreuenden Apothekerinnen Anne Pauly und Carolin Wolf jederzeit gerne zur Verfügung.

Mit freundlichen Grüßen,

Prof. Dr. Johannes Kornhuber
Projektleitung, Direktor der Psychiatrischen und Psychotherapeutischen Klinik

Prof. Dr. Kristina Leuner
Inhaberin der Professur für Molekulare und Klinische Pharmazie

Anne Pauly
Professur für Molekulare und Klinische Pharmazie, Stationsapothekerin

Carolin Wolf
Professur für Molekulare und Klinische Pharmazie, Stationsapothekerin

Pharmazeutische Betreuung psychiatrischer Patienten

Patienten-Einverständniserklärung

Hiermit erkläre ich, ________________________________, an der Studie zur pharmazeutischen Betreuung psychiatrischer Patienten der Psychiatrischen und Psychotherapeutischen Klinik und der Universität Erlangen, Professur für Molekulare und Klinische Pharmazie teilzunehmen.
Ich bestätige, dass ich von der zuständigen Apothekerin __________________________ am __________________ über Ziele und Ablauf, Bedeutung, Vorteile und Risiken der Studie aufgeklärt wurden und eine schriftliche Patienteninformation (Version: 29.06.2012) zur Studie erhalten habe. Eventuell aufgetretene Fragen wurden zufrieden stellend geklärt. Die Aufklärung war für mich in allen Punkten verständlich. Eine Kopie dieser Einverständniserklärung habe ich erhalten.

Für die Entscheidung zur Teilnahme an oben genannter Studie wurde mir ausreichend Zeit eingeräumt.
Ich bin bereit, die an mich ausgegebenen Fragebögen zu allgemeinen Daten zu meiner Person sowie zu den Themen „Symptome und Nebenwirkungen", „Arzneimitteleinnahmeverhalten", sowie gegebenenfalls „auf das Körpergewicht bezogene Lebensqualität" und „Beurteilung des pharmazeutischen Betreuungsservices" gemeinsam mit der betreuenden Apothekerin ordnungsgemäß auszufüllen und die Beratungstermine mit der verantwortlichen Apothekerin wahrzunehmen.

Ich gestatte der zuständigen Apothekerin im Bedarfsfall Kontakt mit meinem Hausarzt bzw. niedergelassenen Psychiater aufzunehmen, um fehlende Angaben zur Medikation zu vervollständigen.

Ich weiß, dass meine Zustimmung zur Teilnahme an oben genannter Studie **freiwillig** ist und **jederzeit und ohne Angabe von Gründen widerrufen** werden kann und dass dies keinen Einfluss auf meine etwaige weitere ärztliche Behandlung hat oder sonstige Nachteile mit sich bringt.

Ich bin damit einverstanden, dass **Mitarbeiter der Apotheke des Universitätsklinikums Erlangen und der Universität Erlangen**, die an dem Projekt zur **pharmazeutischen Betreuung psychiatrischer Patienten** beteiligt sind, Einblick in meine Original-Krankenunterlagen nehmen.

Ich stimme zu, dass Daten, die meine Person betreffen unter der Verantwortung der oben genannten Institutionen **in verschlüsselter Form** verarbeitet und gespeichert sowie anonymisiert für eine mögliche Publikation verwendet werden.

Im Falle des Widerrufs bin ich damit einverstanden, dass meine Daten zu Kontrollzwecken weiterhin gespeichert bleiben. Ich habe das Recht, deren Löschung zu verlangen, sofern gesetzliche Bestimmungen der Löschung nicht entgegenstehen.

____________________ ____________________

Datum Unterschrift
