# Supplementary material for: Effect of a Multi-Dimensional and Inter-Sectoral Intervention on the Adherence of Psychiatric Patients
Source: PLoS One. 2015 Oct 5;10(10):e0139302. doi: 10.1371/journal.pone.0139302 (PMC4593549; doi:10.1371/journal.pone.0139302)
Supplement: S2 File — (PDF) [file pone.0139302.s002.pdf]

To the office
of the ethics committee
of the medicinal department
of FAU Erlangen-Nuremberg
Krankenhausstr. 12
91054Erlangen

Application
For the ethics committee
Of the medicinal department

Please complete in ***German language****,*
Mark with a cross where applicable
For multicenter trials with a pre-vote of a ethic commitee formed according to national laws,
You can use a shortened application form under http://www.ethik.med.uni-erlangen.de *(connection vote)*

Application for evaluation of a research project (no drug trial)

Please submit 9-fold including supplements and one time electronically

**Title of the research project:**
**Pharmaceutical care of stationary psychiatric patients**

I. Project lead

1. Name of the responsible project lead at FAU:
   Prof. Dr. Johannes Kornhuber
   Director of the psychotherapeutical and psychiatric department
   App.-Nr.: 0.853
   Phone: 09131 / 85-34166
   E-Mail: johannes.kornhuber@uk-erlangen.de

   Qualification of the project leader:
   Director of the psychotherapeutical and psychiatric department

2. a) Additional participants on-site:
   Prof. Dr. Kristina Leuner
   Molecular and Clinical Pharmacy
   App.-Nr.: 0.624
   Phone: 09131 / 85-29550
   E-Mail: leuner@pharmtech.uni-erlangen.de

Anne Pauly, pharmacist
Molecular and Clinical Pharmacy
App.-Nr.: 0.632
Phone: 09131 / 85-29575
E-Mail: pauly@pharmtech.uni-erlangen.de

Carolin Wolf, pharmacist
Molecular and Clinical Pharmacy
App.-Nr.: 0.632
Phone: 09131 / 85-29575
E-Mail: cwolf@pharmtech.uni-erlangen.de

b) Additional trial centers (for multi-center trials): none

3. Has this application already been reviewed by the ethics committee of the medicinal department of the Friedrich-Alexander-University Erlangen-Nuremberg?
 yes (please provide serial number) X no

II. Research project

1. planned start of the study: 1st September 2012
Estimated completion: 31st January2014
Duration of participation in the study of individual test persons: 111 days (on average inpatient stay of 21 days as well as follow-up for 3 months after discharge from hospital)

2. Brief outline of the project

The objectives of the planned project aim at collecting data in a clinical study on whether an intensive medical patient's history as well as an intensive consultation of the patient regarding his medication and his illness by integrating a hospital pharmacist into a multidisciplinary treatment team of the psychiatric and psychotherapeutical clinic can lead to an improvement respectively a reduction of the following parameters:

- Number of drug related problems (primary efficacy endpoint)
- Adherence to medication, especially after discharge (primary efficacy endpoint)

Drug related problems are defined as events or circumstances in the patient's drug therapy, which could actually or potentially prevent the achievement of the intended objectives. In this study the focus lies on the following drug related problems: congruence of diagnosis and indication, dosing mistakes, interactions and side-effects, e.g. weight gain and sexual dysfunction.
The WHO defines compliance or commitment to therapy as "The extent to which a patient's behavior - taking medication, following a diet and/or executing lifestyle changes - corresponds with agreed recommendation from a health care provider." Adherence is the extension of the definition of compliance by including the patients' wishes concerning his therapy.
Secondary endpoints are:

- Attitude of the patients towards his medication
- Influence of the patient's attitude towards his medication on medication adherence

Other parameters:

- The patient’s satisfaction with the medical-pharmaceutical care

The planned study on pharmaceutical care is a prospective open interventional study with sequential control and intervention groups. Psychiatric patients from the wards P21 and P31 of the Psychiatric and Psychotherapeutic University Hospital Erlangen are included regardless of the diagnosis.

Further inclusion criteria must be met

- Age ≥ 18
- Capability to fill in questionnaires independently
- Capability to understand spoken and written German
- Capability to give consent
- Inpatient stay in the Psychiatric Clinic Erlangen up to 7 days
- Willingness to get into contact after discharge

Patients are excluded who are being treated only psychotherapeutically und do not take any additional co-medication (non-psychiatric). Patients who are transferred from the protected women’s ward to the participating open wards, are also excluded.

When admitted to hospital, the patient receives a detailed written information about the planned project and a form for the declaration of consent. After a period of reflexion of at least 24 hours, the form is collected and the patient is included in the study or not according to his decision.

To avoid an overlap of supervised and non-supervised patients on the wards, a sequential control group is used: During the first six months (cw 26/12 – cw 09/13) of the study patients are only admitted to the control group, in the following 6 months (cw 14/2013 – cw 39/2013) only to the intervention group. Between the two phases, a wash-out period of 4 weeks takes place in order to guarantee that the patients of the control group have completely finished the hospitalized study phase before beginning the intervention phase.

Pharmaceutical care plan

1 – 2 days after admission to hospital, a thorough medication review including a medical history of all study participants is taken by the pharmacists. Every prescription is examined by means of the “Medication Appropriateness Index [3]. Side effects and adherence of all patients are determined on the basis of questionnaires.

The patients fill in these questionnaires together with the pharmacists one to four days after admission, one day before and three months after discharge. Together with the medication profile the results of these questionnaires are examined on DRP such as missing indication, side-effects or non-adherence. Particular attention is paid to the determination of interactions with the interaction database “Stockley’s”. In the control group, the DRP are only identified and documented (exception for ethical reasons: life-threatening or severely disease worsening complications).

In the intervention group, a solution of the DRP is to be worked out together with a physician.

The adherence in both groups is determined during the hospital stay (1-4 days after admission, and 1-2 days before discharge) and 3 months after discharge. Ten days after admission, the patients of the intervention group receive an intensive consultation concerning their illness and detailed information on their medication, as well as benefit and side effects of the drugs.

The adherence especially being jeopardized after discharge, patients of the intervention group receive a discharge medication plan. Furthermore, these patients are offered a follow-up telephone call by the pharmacist in order to improve adherence for the

following 3 months. During this call, questions are to be asked on change of medication by their physician, the satisfaction with the treatment and the adherence. Open questions and individual problems of the patients with medications can be clarified. If necessary, a thorough consultation is given. Thus, the interface between hospitalized and outpatient treatment is to be closed by pharmaceutical care.

An overview of the timing of the study can be found in annex Nr. 1

3. **Study-related acitvities:**

Please describe all measurements which are carried through on a study related basis as well as all essential deviations from customary treatment.

In order to determine a.m. parameters, the patients of the intervention- and control group fill in the following validated questionnaire together with the pharmacist on the day of admission, 1-2 days before discharge, and 3 months after discharge during the follow-up phase.

**Questionnaire**		**Parameter**
**UKU**	The Udvalg for Kliniske Undersøgelser Side Effect Rating Scale, nach Lingjaerde et al	Side-effects of psychotropic drugs
**MARS**	Medication Adherence Report Scale- German version (Mahler et al)	Adherence
**DAI**	Drug Attitude Inventory (Hogan et al)	Adherence and attitude
**Beurteilung des pharmazeutischen Betreuungsservices (BphB, intervention group)**	Evaluation of the pharmaceutical care service	Satisfaction with pharmaceutical care

The pharmacists fill in the “Medication Appropriateness Index” as a guideline in order to identify drug related problems and to compare admission- and discharge medication in respect to appropriateness of the therapy.

Furthermore, a consultation on medication and the diagnosed illnesses for those of the intervention group is offered. The consultation consists of detailed information on

- Psychiatric disease of the patient
- Accompanying diseases (hypertension, diabetes mellitus)
- Which medication for which disease is indicated
- Dosage- and taking regime

The securing of adherence when patients are discharged from hospital into the outpatient sector is paramount (seamless care)

The patient satisfaction concerning the pharmaceutical care is judged by a questionnaire developed for a study of pharmaceutical care with bypass patients. [9]

4. Is the study carried through according to the Declaration of Helsinki of 1996, revised by the 48. General Assembly of the World Medical Association?

YES

Please specify if all other trial options have been exercised.
No other trial options are known.

5. Nature of the research project
Does it concern
 a diagnostic examination
 a therapeutic examination
 a compatibility test
 an excludingly scientific trial
X a study on care research

6. Legal regulations
a) Is it a study with the objective to research or to prove clinical or pharmacological impacts /effects) of drugs or to detect side effects or investigate the absorption, distribution, metabolism or excretion with the aim of being convinced of the quality, safety and efficacy of the medication?

No, pharmaceutical care study.

b) Is it a clinical trial according to § 20 Medizinproduktegesetz (MPG)?
 yes X no

Please give reasons. Is there a CE Certificate for the medical product? Are there also invasive or other burdening examination to be carried through?

c) Is it a project according to § 8 of the law for regulation of transfusion management?
 yes X no

7. Is it a trial according to
§ 23 Strahlenschutzverordnung? yes X no
§ 28 Röntgenverordnung? yes X no

Please give reasons. IF yes, will necessary scientific advice be sought?

8. Type of the study:
X open
 blind
 double-blind
X comparative
 randomized
 multicenter
 field study
X pilot study

9. Scientific outline of the study, in particular:

a. Explanation of the aim of the experiment

Primary aim of the study is the reduction of DRP as well as the improvement of adherence by providing pharmaceutical care in the inpatient setting and especially 3 months after discharge.

b. Description of the current state of knowledge

In Germany, the 12-month-prevalence of psychiatric disorders is 37 % for women and 25,3 % for men [4]. In recent years this number has increased, especially with depressive disorders [5]. It is extremely important to get an exact and complete medication history of the drug therapy of these patients, because psychiatric disorders are often accompanied by comorbidity and polypharmacy [6]. The risk of DRP rises exponentially. Furthermore, the rate of therapy discontinuation is very high due to the special mode of action of the drugs (onset of effect after 2 -3 weeks, but occurrence of side effects immediately). The number of adherent patients varies from 20 to 70 % even after the introduction of modern preparations [2]. Adherence is a very important component of an adequate and successful therapy of psychiatric disorders. The risk of rehospitalization of a non-adherent schizophrenic patient is according to Valenstein et al 2.4 times higher compared to an adherent schizophrenic patient [7].. The same applies to patients with affective disorders. Discontinuation of therapy of patients with affective disorder leads to an increase of 77 % in the risk of relapse [8].

The concept of the pharmaceutical care is the basis for reduction of DRP as well as for the improvement of adherence. Pharmaceutical care is based on the intensive cooperation of the patient, the doctor in charge and the pharmacist. The aim is to instruct the patient together with the doctor in order to apply the medication securely and appropriately, to recognize and to solve possible DRP, to optimize the drug therapy and resulting from this, to improve the patient's quality of life.

A Dutch working group with Joanna Klopotowska examined the impact of a ward pharmacist on the number of DRP in an intensive care unit. A reduction of DRP from 190.5 per 1000 observed patient days down to 62.5 per 1000 days could be demonstrated [9]. A study on pharmaceutical care of patients with coronary heart disease proved a significant increase in adherence after an aortocoronary bypass surgery. After 12 months, adherence in the intervention group was 90 % (base value 88 %) and in the control group 71 % (base value 81 %). The care concept included apart from talks with a hospital pharmacist during the hospital stay also follow-up talks during the outpatient phase as well as the handing out of written information [10].

The present study on pharmaceutical care of psychiatric patients in hospital is to investigate if similarly positive effects on the number of DRP as well as on the adherence of medication in this group of patients can be achieved. Validated questionnaires are used for quantification.

To our knowledge no comparable studies with psychiatric patients exist. A first project in Great Britain covered 282 interventions by pharmacists in ward [11]. In a study on pharmaceutical care in the USA, 62 % of the patients experienced a more than 30 % improvement in the Brief Psychiatric Rating Scale [12]. In another study, an improvement of 19% in adherence was achieved [13]. With the present, planned study, for the first time, pharmaceutical care by pharmacists in ward in close collaboration with a clinician is to be implemented in a German psychiatric hospital.

Additionally, the benefit of the given care of a pharmacist has to be quantitatively evaluable.

10. **Information on the risk-benefit-relation**
a) Which benefit is to be expected of the results of the study?
aa) A thorough medication history as well as medication review can prevent DRP such as double prescription, interactions and unnecessary drug intake in order to make the treatment regime of the patient as effective and simple as possible. Moreover, the therapy success, i.d. the remission of the disorder, is influenced significantly by the patient's adherence over a long period. Aim of the project is to improve the understanding of medication and disease by consequently training and informing about proper pharmaceutical drug usage in order to increase adherence. If possible, retard drugs will replace multiple doses, in order to simplify the patient's therapy scheme. The patient's satisfaction with the treatment is to be improved by informing on avoidance strategies concerning possible side effects and on treatment respectively handling of actually occurring side effects.

ab) for the medicine
Double prescriptions, prescriptions without indication as well as side effects of drugs caused by interactions can be prevented by a targeted medication review and thereby medicine-related cost can be saved. Furthermore a long-termed therapy success leads to a reduced recurrence rate and hence to a reduced rate of rehospitalization. Thus, pharmaceutical care presents in case of positive results an approach to improve the quality of care as well as to use resources more efficiently.

ac) for the science (e.g results which do not serve directly therapeutical purposes)
It is for the first time that in Germany a pharmacist in a psychiatric ward tries to reduce DRP by pharmaceutical care and to improve the patient's adherence in order to support therapy success. Due to the design of the study with control groups, a reduction of drug related problems compared to standard care can be shown for the first time.

b) What are the risks of the study for the trial participants?
ab)What kind of risks? Risk assessment, foreseeable risks of the care and other study related procedures which are used (including pain, inconveniences, complaints, violation of the personal integrity and measures to avoid and/or to treat unforeseeable/unwanted incidents.
The study does not involve any risks.

bb) How high is the probability that the risks are realized? How safely is the probability assessed?

c) Why is the possible risk in relation to the expected benefit justifiable in your opinion?
/

d) Will interim results be analyzed in order to recognize a trend?
X yes ☐ n0

e) Have criteria for changing or stopping the trial been defined?
☐ yes, which? X no

11. For clinical trials according to §§ 20-24 MPG:

/

Please provide according to § 3 Abs. 1-3 of the regulation about clinical trials of medical devices and of changes of laws for medical devices the necessary supplements. The current version is provided under [*http://www.ethik.med.uni-erlangen.de*](http://www.ethik.med.uni-erlangen.de)

12. a) Is a statistician involved? X yes ☐ no
b) Which statistical methods shall be used?
With the exception of the patient's satisfaction with the pharmaceutical care (only descriptive) average values resp. median, value ranges, standard deviation and confidence interval will be specified for all measured values (primary and secondary end points). In order to determine statistically important differences, for continuous measured values, T-test will be carried when values are presumably normally spread, the Mann-Whitney-U-Test, when presumably values are not normally spread. In the case of binary values the Chi-Quadra-Test will be performed.

13. a) Is it a multi-centric study (i.e. a trial conducted according to a single protocol, which is carried out at more than one site and therefore by more than one investigator)?
☐ yes X n0
b) Have there been or are there trials conducted with the same aim?
☐ yes, where? X no

14. Who has initiated the study?
Department of Psychiatry and Psychotherapy and the team of Molecular & Clinical Pharmacy. .

15. How is the study being financed? *(Please state if third party funds from non-public sector have been applied for? If yes, how much?)*
The study is developed and performed within two PhD projects.

16. The expense allowance is paid for by (please name contact persons)
Molecular & Clinical Pharmacy,.(Prof. Dr. Kristina Leuner)

III. Information on trial participants

1. Number (*please specify division into groups in comparing studies)*
Inclusion of 123 patients in the control and the intervention group each

For null hypothesis based studies:
Has a sample size estimation been made?
X yes ☐ no

2. Age and Gender *(please provide the age of the trial participants as well as upper and lower limits intended as exclusion criteria)*
Age: 18 - ∞ , male and female

3. Status: Are the trial participants

- [ ] healthy persons
- [ ] pregnant or breast-feeding women
- [ ] children or adolescents

X relevantly sick people with psychiatric disorders according to ICD-10

- [ ] persons suffering from other diseases? (Especially psychiatric disorders, which give rise to doubt regarding cognitive faculty and legal capacity)

4. Which other inclusion criteria are established? (e.g. co-medication)
   - age: ≥ 18
   - capability to fill in questionnaires independently
   - capability to understand written and spoken German
   - capability for consent
   - inpatient stay in the psychiatric hospital in Erlangen > 7 days
   - willingness for contact after discharge

5. Which other exclusion criteria (e.g. end stage renal or liver insufficiency, forbidden co-medication)
   - Exclusively psychotherapeutic care without any co-medication
   - Transfer from the closed women's ward P12 to the open wards

6. Shall people participate who are put away in an institution by official or court law?

   ☐ yes X no

7. Shall people participate who have already participated on other research project?

   X yes ☐ no

   How long ago must the latest participation be? /

8. For Studies with minors (or other not legally competent persons)

   a. Why can the study not be conducted with adults?

   b. Are information and consent guaranteed?
   (*please provide explanation*)

   ☐ yes ☐ no, because

   c. Are additional information and consent of minors (not fully legally competent) guaranteed, who themselves are capable of understanding the nature, the importance and the consequence of the trail and to determine their wishes accordingly?

   ☐ yes ☐ no

9. Proband's insurance
   Will an insurance in favor of the trial participants be taken out?

   ☐ yes (please add policy quoting the insurance company and the amount of insurance benefit.*)*

   X no

10. Confidentiality/Data protection

Are medical confidentiality and data protection regulations observed?

YES

11. Payment for test persons

Shall the trial participants be paid a compensation (e.g. for expenses)

☐ yes, in the amount of…… EUR X no

12. In what way shall trail participants be **informed** about nature, significance and consequences of the study?

Please add in German:
Documentation of the content of the patient's education by the doctor in charge (data sheet), especially referring to:

- Aim and methods of the study
- Benefits and risks of the study
- Known and possibly anticipated effects and side effects of drugs
- Operations made only for scientific reasons
- Appropriate behavior of the patients during and after the trial
- Revocability of consent
- Exclusion criteria (e.g. pregnancy/breast feeding)
- Name and telephone number of local contact persons

Is added (Supplement Nr. 2)!

13. In what way shall participants declare their consent to take part in the study? *(please add a formulated German statement including data protection consent declaration)*

Is added (Supplement Nr. 3)!

Literature

1. Foppe van Mil, J.W., et al., *Drug-related problems in public pharmacies.* Arzneimittelbezogene probleme in der öffentlichen apotheke, 2001. **146**(16): p. 24-30.
2. World-Health-Organization, *Adherence to long-term therapies: evidence for action.* . Geneva, 2003.
3. Hanlon, J.T., et al., *A method for assessing drug therapy appropriateness.* Journal of Clinical Epidemiology, 1992. **45**(10): p. 1045-1051.
4. Jacobi, F., M. Klose, and H.U. Wittchen, *[Mental disorders in the community: healthcare utilization and disability days].* Bundesgesundheitsblatt Gesundheitsforschung Gesundheitsschutz, 2004. **47**(8): p. 736-44.
5. Bühren, A., et al., *Mental disorders: All specialties are needed.* Psychische erkrankungen - Alle fachgebiete sind gefordert, 2008. **105**(17): p. A880-A884.
6. Kampfhammer, H., *Psychische Störungen bei somatischen Krankheiten.* Psychiatrie, Psychosomatik und Psychotherapie, Springer-Verlag, 2011. **4. Auflage.**
7. Valenstein, M., et al., *Pharmacy data identify poorly adherent patients with schizophrenia at increased risk* [illegible]

Anhänge:

1. Studienplan
2. Patienteninformation
3. Einverständniserklärung

Kurzfassung

Erfassungsphase: Erfassung der Gegebenheiten, Durchführbarkeit, Prüfung Fragebogen, Einschlusskriterien
Dauer: 3 Monate

Studienphase: pharmazeutische Betreuung auf den Stationen P21 und P31
Dauer: 12 Monate

**Zeit**		**Arzneimittelbezogene Probleme**		**Adherence**
**1. Kontrollgruppe: 6 Monate**				
Tag X		Aufnahme		Aufnahme
Tag X + 1-4 Tage		1.Visite und Anamnese des Apothekers, keine Intervention		1. Visite des Apothekers, keine Intervention, MARS-DAI, UKU, evtl. IWQOL-lite, Gewicht
Tag A + 10 Tage		2. Visite des Apothekers, keine [illegible]		3. Visite des Apothekers, keine Intervention, [illegible]

Pharmazeutische Betreuung psychiatrischer Patienten

Patienteninformation

Liebe Patientin, lieber Patient,

Sie wurden vor kurzem in der Psychiatrischen und Psychotherapeutischen Klinik des Universitätsklinikums Erlangen aufgenommen. Wir hoffen, dass Sie sich hier wohlfühlen und bald Besserung finden.

Die Psychiatrische Klinik der Universität Erlangen und die Professur für Molekulare und Klinische Pharmazie führen seit März 2012 ein Projekt zur pharmazeutischen Betreuung psychiatrischer Patienten unter Leitung von Herrn Prof. Kornhuber, Direktor der Psychiatrischen und Psychotherapeutischen Klinik, durch. Dabei wollen wir klären, ob es sinnvoll ist, psychiatrische Patienten neben ihrer bisherigen Therapie zusätzlich durch einen Apotheker zu ihrer Arzneimittelanwendung zu informieren und strukturiert zu betreuen.

Hintergründe und Ziele des Projektes:

Der Erfolg einer psychiatrischen Therapie hängt in hohem Maße von Ihrer Mitarbeit ab. So kann ein Medikament seine Wirkung nur entfalten, wenn es von Ihnen als Patient regelmäßig und richtig eingenommen wird. Ein Ziel unseres Projektes ist es daher, durch unsere pharmazeutische Betreuung Ihre sichere und gute Arzneimittelanwendung zu fördern und gemeinsam mit Ihnen und den behandelnden Ärzten zu einem verbesserten Therapieergebnis beizutragen.

Eine gründliche Überprüfung Ihrer Medikation durch eine Apothekerin kann eventuell auftretende Probleme wie z.B. Wechsel- oder Nebenwirkungen frühzeitig aufdecken und damit verhindern. Als zweites Ziel möchten wir somit erreichen, dass Ihre Arzneimitteltherapie noch sicherer und angenehmer wird.

Langfristig soll durch die intensive Zusammenarbeit zwischen Arzt, Pflege und Apotheker die Qualität der Arzneimitteltherapie im Krankenhaus verbessert werden.

Die beschriebene Studie dient *nicht* dazu, neue Arzneimittel zu testen.

Ablauf des Projektes:

Die Betreuungsdauer für alle Projektteilnehmer erstreckt sich über den gesamten Klinikaufenthalt. Die ambulante Nachbetreuung endet 3 Monate nach Entlassung. Um die Einflüsse der zusätzlichen Betreuung durch einen Apotheker untersuchen zu können, werden die Teilnehmer der Studie nach Aufnahmedatum in folgende zwei Gruppen eingeteilt:

Gruppe 1 (September 2012 – Februar 2013):

Patienten dieser Gruppe erhalten die übliche Betreuung durch das ärztliche und pflegerische Team der Stationen. Während Ihres stationären Aufenthaltes sowie im Rahmen des Gesprächs 3 Monate nach Entlassung aus dem Krankenhaus erhalten Sie drei Fragebögen zu den Themen „Symptome und Nebenwirkungen“, „Arzneimitteleinnahmeverhalten“ und gegebenenfalls „auf das Körpergewicht bezogene Lebensqualität“. Diese werden gemeinsam mit der betreuenden Apothekerin ausgefüllt. Außerdem wird Ihr Medikationsprofil von einer Apothekerin aufgenommen.

Gruppe 2 (April 2013 – September 2013):
Patienten dieser Gruppe erhalten neben der üblichen Betreuung durch das ärztliche und pflegerische Team der Station individuelle Beratungsgespräche sowie eine gründliche Überprüfung des Medikationsprofils durch eine Apothekerin. Sie erhalten eine Woche und drei Monate nach Entlassung einen Termin für ein weiteres Beratungsgespräch mit der betreuenden Apothekerin.
Während Ihres stationären Aufenthaltes sowie im Rahmen der Gespräche nach Entlassung erhalten Sie drei Fragebögen zu den Themen „Symptome und Nebenwirkungen", „Arzneimitteleinnahmeverhalten", „Lebensqualität" sowie einmalig den Fragebogen „Beurteilung des pharmazeutischen Betreuungsservice". Diese Fragebögen werden gemeinsam mit der betreuenden Apothekerin ausgefüllt. Auf Grundlage des Medikationsprofils und der Fragebögen möchten wir die auftretenden Probleme der Patienten mit ihrer Therapie in Zusammenarbeit mit Arzt und Pflege lösen.

Risiken und Nutzen des Projektes:
Die ärztliche Therapie im Rahmen Ihres Aufenthaltes in der Psychiatrischen Klinik wird durch dieses Projekt in keiner Weise beeinflusst. Es entstehen keine zusätzlichen Risiken für Sie. Die endgültige Entscheidung der Arzneimitteltherapie liegt unverändert beim behandelnden Arzt.
Ihre Arzneimitteleinnahme soll für Sie so einfach, angenehm und sicher wie möglich gestaltet werden. Dadurch sollen Therapietreue und Therapieerfolg verbessert sowie eventuell auftretende Probleme reduziert werden.

Datenschutz:
Im Laufe der Studie werden patientenbezogene Daten von Ihnen erfasst. Dabei handelt es sich neben allgemeinen Angaben zu Ihrer Person zum einen um die Dokumentation Ihrer Medikation und zum anderen um Daten aus den von Ihnen ausgefüllten Fragebögen und den mit Ihnen geführten Beratungsgesprächen. Alle anfallenden Daten werden nur verschlüsselt gespeichert und ausgewertet sowie anonymisiert für eine mögliche Publikation verwendet. Die personalisierten Daten werden Dritten nicht zugänglich gemacht.

Rechte der Teilnehmer:
Die Teilnahme an der oben beschriebenen Studie ist freiwillig. Sollten Sie die Teilnahme an der Studie ablehnen, ist Ihr weiterer Therapieablauf in der Psychiatrischen und Psychotherapeutischen Klinik natürlich in keiner Weise beeinträchtigt.
Wenn Sie im Laufe der Studie Ihre Einwilligung zurückziehen möchten, ist dies jederzeit und ohne Angabe von Gründen möglich. Es entstehen Ihnen hierdurch selbstverständlich keinerlei Nachteile.

Wir hoffen, dass wir Ihr Interesse an unserer Studie zur pharmazeutischen Betreuung psychiatrischer Patienten geweckt haben. Wir würden uns freuen, Sie als Teilnehmer/in begrüßen zu dürfen. Zu diesem Zweck ist es notwendig, beiliegende Einverständniserklärung zu unterzeichnen.

Wenn Sie noch weitere Fragen zu unserem Projekt oder dessen Ablauf haben, stehen Ihnen Ihre betreuenden Apothekerinnen Anne Pauly und Carolin Wolf jederzeit gerne zur Verfügung.

Mit freundlichen Grüßen,

Prof. Dr. Johannes Kornhuber<br>Projektleitung, Direktor der Psychiatrischen und Psychotherapeutischen Klinik	Prof. Dr. Kristina Leuner<br>Inhaberin der Professur für Molekulare und Klinische Pharmazie	Anne Pauly<br>Professur für Molekulare und Klinische Pharmazie, Stationsapothekerin	Carolin Wolf<br>Professur für Molekulare und Klinische Pharmazie, Stationsapothekerin

Pharmazeutische Betreuung psychiatrischer Patienten

Patienten-Einverständniserklärung

Hiermit erkläre ich, ________________________________, an der Studie zur pharmazeutischen Betreuung psychiatrischer Patienten der Psychiatrischen und Psychotherapeutischen Klinik und der Universität Erlangen, Professur für Molekulare und Klinische Pharmazie teilzunehmen.
Ich bestätige, dass ich von der zuständigen Apothekerin __________________________ am ___________________ über Ziele und Ablauf, Bedeutung, Vorteile und Risiken der Studie aufgeklärt wurden und eine schriftliche Patienteninformation (Version: 29.06.2012) zur Studie erhalten habe. Eventuell aufgetretene Fragen wurden zufrieden stellend geklärt. Die Aufklärung war für mich in allen Punkten verständlich. Eine Kopie dieser Einverständniserklärung habe ich erhalten.

Für die Entscheidung zur Teilnahme an oben genannter Studie wurde mir ausreichend Zeit eingeräumt.
Ich bin bereit, die an mich ausgegebenen Fragebögen zu allgemeinen Daten zu meiner Person sowie zu den Themen „Symptome und Nebenwirkungen“, „Arzneimitteleinnahmeverhalten“, sowie gegebenenfalls „auf das Körpergewicht bezogene Lebensqualität“ und „Beurteilung des pharmazeutischen Betreuungsservices“ gemeinsam mit der betreuenden Apothekerin ordnungsgemäß auszufüllen und die Beratungstermine mit der verantwortlichen Apothekerin wahrzunehmen.

Ich gestatte der zuständigen Apothekerin im Bedarfsfall Kontakt mit meinem Hausarzt bzw. niedergelassenen Psychiater aufzunehmen, um fehlende Angaben zur Medikation zu vervollständigen.

Ich weiß, dass meine Zustimmung zur Teilnahme an oben genannter Studie **freiwillig** ist und **jederzeit und ohne Angabe von Gründen widerrufen** werden kann und dass dies keinen Einfluss auf meine etwaige weitere ärztliche Behandlung hat oder sonstige Nachteile mit sich bringt.

Ich bin damit einverstanden, dass **Mitarbeiter der Apotheke des Universitätsklinikums Erlangen und der Universität Erlangen**, die an dem Projekt zur **pharmazeutischen Betreuung psychiatrischer Patienten** beteiligt sind, Einblick in meine Original-Krankenunterlagen nehmen.

Ich stimme zu, dass Daten, die meine Person betreffen unter der Verantwortung der oben genannten Institutionen **in verschlüsselter Form** verarbeitet und gespeichert sowie anonymisiert für eine mögliche Publikation verwendet werden.

Im Falle des Widerrufs bin ich damit einverstanden, dass meine Daten zu Kontrollzwecken weiterhin gespeichert bleiben. Ich habe das Recht, deren Löschung zu verlangen, sofern gesetzliche Bestimmungen der Löschung nicht entgegenstehen.

____________________ ____________________

Datum Unterschrift
